# Ventrolateral Periaqueductal Gray Neurons Are Active During Urination

**DOI:** 10.3389/fncel.2022.865186

**Published:** 2022-06-23

**Authors:** Yu Rao, Ziyan Gao, Xianping Li, Xing Li, Jun Li, Shanshan Liang, Daihan Li, Jinliang Zhai, Junan Yan, Jiwei Yao, Xiaowei Chen

**Affiliations:** ^1^Brain Research Center and State Key Laboratory of Trauma, Burns, and Combined Injury, Third Military Medical University, Chongqing, China; ^2^Center for Neurointelligence, School of Medicine, Chongqing University, Chongqing, China; ^3^School of Physical Science and Technology, Guangxi University, Nanning, China; ^4^Medical College, Guangxi University, Nanning, China; ^5^Department of Urology, Southwest Hospital, Third Military Medical University, Chongqing, China; ^6^Guangyang Bay Laboratory, Chongqing Institute for Brain and Intelligence, Chongqing, China

**Keywords:** fiber photometry, pontine micturition center, ventrolateral periaqueductal gray (vlPAG), urination, cystometry, rabies virus

## Abstract

The ventrolateral periaqueductal gray (VLPAG) is thought to be the main PAG column for bladder control. PAG neurons (especially VLPAG neurons) and neurons in the pontine micturition center (PMC) innervating the bladder detrusor have anatomical and functional synaptic connections. The prevailing viewpoint on neural control of the bladder is that PAG neurons receive information on the decision to void made by upstream brain regions, and consequently activate the PMC through their direct projections to initiate urination reflex. However, the exact location of the PMC-projecting VLPAG neurons, their activity in response to urination, and their whole-brain inputs remain unclear. Here, we identified the distribution of VLPAG neurons that may participate in control of the bladder or project to the PMC through retrograde neural tracing. Population Ca2^+^ signals of PMC-projecting VLPAG neurons highly correlated with bladder contractions and urination as shown by *in vivo* recording in freely moving animals. Using a RV-based retrograde trans-synaptic tracing strategy, morphological results showed that urination-related PMC-projecting VLPAG neurons received dense inputs from multiple urination-related higher brain areas, such as the medial preoptic area, medial prefrontal cortex, and lateral hypothalamus. Thus, our findings reveal a novel insight into the VLPAG for control of bladder function and provide a potential therapeutic midbrain node for neurogenic bladder dysfunction.

## Introduction

Urine storage and elimination are two major physiological functions of the bladder, which are controlled by complex neural circuits distributed throughout the brain, spinal cord, and peripheral nerves (Fowler et al., 2008; Xiao et al., 2022). However, abnormalities and lesions at any level of this control system can lead to neurogenic lower urinary tract symptoms (LUTS; Benarroch, 2010; Tish and Geerling, 2020). The most troublesome LUTS are those affecting bladder control, such as urgent urination, urinary frequency, urinary incontinence, and difficulty urinating (Griffiths, 2004). There is a lack of safe and effective treatments for disorders involving decreased bladder control (McDonough and Ryan, 2016). A major cause of the clinical dilemmas identified above is a limited understanding of the underlying neural mechanisms of normal bladder function (Kitta et al., 2015; Tish and Geerling, 2020). Breakthrough research focused on neural control of the bladder will improve the diagnosis and treatment of neurogenic bladder dysfunction.

Periodic elimination of urine (voiding) is generated by a spino-bulbospinal reflex circuit (Griffiths and Fowler, 2013; de Groat et al., 2015). The source of the voiding reflex is the pontine micturition center (PMC) located in the brainstem (Fowler et al., 2008; Benarroch, 2010). The formation of lesions in the PMC and deletion of PMC glutamatergic neurons can result in aberrant urinary characteristics, such as retention of urine and overflow incontinence (Lachkepiani et al., 2011; Verstegen et al., 2019). Electrical stimulation and optogenetic activation of PMC neurons induce bladder contractions and urination (Sugaya et al., 1987; Verstegen et al., 2019). Functional neuroimaging and fiber photometry recording studies show that PMC neurons become active during urination and bladder contraction (Fowler and Griffiths, 2010; Verstegen et al., 2019; Yao et al., 2019). Recent studies report that PMC neurons that express corticotropin releasing hormone (CRH) project to the sacral parasympathetic neurons (bladder motor neurons) at the sacral parasympathetic nuclei (SPN) to control bladder contraction (Hou et al., 2016). In the periphery nervous system, neurons in the major pelvic ganglion (MPG) receive parasympathetic preganglionic fibers from neurons at the SPN and then send their postganglionic nerves to the bladder directly for supplying parasympathetic innervation (Fowler et al., 2008; Arellano et al., 2019). However, PMC neurons that express estrogen receptor 1 (ESR1) project to the lumbosacral dorsal gray commissure (DGC) to relax the urethral sphincter (Keller et al., 2018). Therefore, the PMC is an essential command center for the voiding reflex.

In addition to the PMC, the periaqueductal gray (PAG) has also received more attention as an important region for controlling the bladder (Zare et al., 2019). A previous clinical report has shown that a small lesion in the PAG causes decreased bladder sensation and urinary retention (Yaguchi et al., 2004). Electrical or chemical stimulation at some sites of the PAG elicits bladder contractions or blocks reflex bladder contractions (Taniguchi et al., 2002; Matsumoto et al., 2004). Functional brain imaging in humans and in rats demonstrates that the PAG is active during urine storage (Athwal et al., 2001) and displays more intense activity during voiding (Tai et al., 2009). Neural tracing experiments suggest that spinal neurons in the lumbosacral dorsal horn receiving afferent signals from the bladder project directly to PAG neurons (Klop et al., 2005; Kuipers and Klop, 2006) and the PAG sends projections to the PMC (Blok and Holstege, 1994; Kuipers et al., 2006). Thus, it is proposed that the PAG also serves as a critical role in urination control.

The PAG is divided into four columns, including the lateral column (LPAG), the ventrolateral column (VLPAG), the dorsomedial column (DMPAG), and the dorsolateral column (DLPAG) (Zare et al., 2019). It has been proven that every column performs different functions (Bandler and Shipley, 1994; Zare et al., 2019). Among them, the VLPAG is thought to be the major PAG column for bladder control (Zare et al., 2019). Animal experiments confirm that chemical or electrical stimulation in the VLPAG increases the frequency of urination and bladder contraction (Matsuura et al., 2000; Taniguchi et al., 2002; Stone et al., 2015). Chemical inhibition and lesion of the VLPAG suppress voiding and impair bladder contraction (Matsuura et al., 1998; Liu et al., 2004; Stone et al., 2011). VLPAG neurons are labeled with retrograde tracers that are injected into the PMC (Verstegen et al., 2019). Optogenetic activation of VLPAG neuron axon terminals in the PMC also induces urination and bladder contractions (Verstegen et al., 2019). Taken together, the VLPAG may control urination or bladder contraction through its direct projection to the PMC. To the best of our knowledge, the exact location of VLPAG neurons projecting to the PMC, as well as their activity in response to urination and whole-brain inputs of these VLPAG neurons, remains unclear.

Therefore, in the current study, we investigated the anatomical location of VLPAG neurons innervating the bladder detrusor using pseudorabies virus-mediated trans-multisynaptic tracing. We also performed rabies virus-based trans-synaptic tracing to examine the distribution of VLPAG neurons that project to the PMC. We established a stable virus labeling strategy to specifically label VLPAG neurons projecting to the PMC. In order to record urination-related activities of VLPAG neurons projecting to the PMC, we used *in vivo* fiber photometry and cystometry simultaneously in freely moving animals. We determined that VLPAG neurons projecting to the PMC correlated with urination and bladder contraction. We further uncovered that VLPAG neurons projecting to the PMC received extensive inputs from higher brain regions. Thus, our findings identify the exact location of urination-related VLPAG neurons and indicate that the VLPAG is involved in controlling bladder function.

## Materials and Methods

### Animals

All experimental manipulations were performed in accordance with the Animal Welfare Guidelines of Third Military Medical University (TMMU) and the National Institutes of Health Guidelines for the Care and Use of Laboratory Animals and were approved by the TMMU Animal Care and Use Committee. Wild-type C57BL/6J mice and CRH-IRES-Cre (Cat# 12704) transgenic mice originated from the Laboratory Animal Center of TMMU or Jackson Laboratory were utilized in this study. All animals were group-housed (four to five individuals per cage) at 20–25°C under 12-h light/dark cycle conditions with food and water readily available. Adult CRH-Cre mice that were 8–20 weeks of age were utilized in RV-based retrograde tracing experiments. The animals used in the other experiments were adult C57BL/6J mice. Animals implanted with optical fibers for fiber photometry recordings were housed individually. Animal numbers of each experiment are indicated in each figure legend.

### PRV Retrograde Trans-multisynaptic Tracing From the Bladder

For PRV retrograde tracing experiments, adult C57BL6/J mice were anesthetized with 10 ml/kg sodium pentobarbital (1%). The bladder was accessed through a midline incision in the lower abdomen. Using a glass micropipette (20 μm diameter of the tip) connected with injection pump, 1.5 μl PRV531-EGFP (2.00 × 10^9^ PFU/ml, BrainVTA Technology Co., Ltd., Wuhan, China) was injected into the dome of the bladder at a speed of 300 nl/min. After being held in the injection region for 5–10 min, the micropipette was slowly pulled out to avoid the virus leaked from the bladder detrusor. The injection site was disinfected with alcohol cotton balls and the surgical wound was sutured. Postoperative mice were sent back to their cages and were perfused before showing signs of death.

### Stereotaxic Injection and Fiber Implantation

Animals received isoflurane (4% induction, 1%–1.5% maintenance throughout the surgery) for anesthesia and were then stabilized in a stereotaxic frame (RWD Technology Corp., Ltd.; Shenzhen, China) with a heating pad for temperature support. Animals were considered to be kept at a sufficient depth of anesthesia, characterized by no response to tail pinch and the loss of pedal withdrawal reflex. After local application of 100 μl lidocaine hydrochloride (2%) under the skin, a midline scalp incision was made by using eye scissors to expose the skull. The craniotomy (1 mm × 1 mm) above each target brain region was drilled using a dental drill (0.5 mm in diameter). Different types of viruses were delivered into the PMC or the VLPAG through pressure injection using glass micropipette attached to a syringe (1 ml). The injection speed was about 20 nl/min. After each injection, the micropipette was left at the injection point for 10 min before withdrawal. The scalp wound was sutured. Then, animals were put back to their home cages and allowed to recover for 3 weeks prior to optic fiber implantation. The coordinates for target injection areas included the PMC (AP, −5.45 mm; ML, −0.7 mm; DV, −3.14 mm from dura) and the VLPAG (AP, −4.72 mm; ML, −0.5 mm; DV, −2.10 mm from dura), as determined by the mouse brain atlas (Paxinos and Franklin Mouse Brain Atlas, second edition).

For fiber photometry experiments, 100 nl volume of AAVRetro-hSyn-Cre (AAV2/2, titer: 10.0 × 10^12^ vg/ml, vector genome per ml, TaiTool Bioscience Co., Ltd., Shanghai, China) mixed with CTB555 (invitrogen, C34776) was unilaterally injected into the PMC. Then, 100 nl of AAV-Dio-GCaMP6f (AAV2/9, titer: 0.5 × 10^12^ vg/ml, Obio Biotechnology Co., Ltd. Shanghai, China) was injected into the ipsilateral VLPAG of the experimental group wild-type C57BL/6J mice, whereas 100 nl of AAV-Dio-EGFP (AAV2/9, titer: 4.20 × 10^12^ vg/ml, BrainVTA Technology Co., Ltd., Wuhan, China) was unilaterally injected into the ipsilateral VLPAG of the control group wild-type C57BL/6J mice. A 200 μm diameter optic fiber (NA 0.48, Doric lenses, Quebec City, QC, Canada) that was fastened with a small metal cannula (ID. 0.51 mm, OD. 0.82 mm) was implanted 50 μm above viral injection region of the VLPAG three weeks after AAVs injection. Then, the optic fiber was attached to each animal’s skull using dental cement as an adhesive. Finally, these animals rested for 3–5 days prior to GCaMP6f detection during urination events.

### RV Retrograde Tracing

To label the upstream PAG neurons of PMC^CRH^ neurons, we first injected about 50 nl of a mixture containing 1:1 volumes of AAV-Dio-EGFP-TVA (AAV2/5, titer: 2.00** ×** 10^12^ vg/ml, BrainVTA Technology Co., Ltd., Wuhan, China) and AAV-Dio-RVG (AAV2/5, titer: 2.00 × 10^12^ vg/ml, BrainVTA Technology Co., Ltd., Wuhan, China) into the unilateral PMC of CRH-Cre mice. After waiting three weeks for these AAVs expression, 100 nl of RV-EnVA-ΔG-DsRed (titer: 2.00 × 10^8^ IFU/ml, BrainVTA Technology Co., Ltd., Wuhan, China) was stereotaxically injected into the same area of these CRH-Cre mice. To explore the upstream brain areas of PMC-projecting VLPAG neurons, 100 nl volume of AAVRetro-hSyn-Cre (mixed with CTB555) was injected into the right PMC and 50 nl of the 1:1 volume mixture of AAV-Dio-EGFP-TVA and AAV-Dio-RVG was administered into the right VLPAG of wild-type mice of a C57BL/6J background at the same time. Three weeks later, 100 nl of RV (RV-EnvA-ΔG-DsRed) was injected into the same site of the VLPAG of these wild-type C57BL/6J mice. These mice were perfused on day 7 after RV injection.

### Fiber Photometry Recordings in Freely Moving Mice

Fiber photometry recordings were performed as described previously (Zhang et al., 2017; Yao et al., 2018, 2019). The fiber photometry system (FOM-02M FiberOptoMeter, Suzhou Institute of Biomedical Engineering and Technology, China) was used for measuring population neuronal activity. In order to decrease photo bleaching, light power at the tip of the optic fiber was adjusted to 0.22 mW/mm^2^. Before recordings of PMC-projecting VLPAG neurons activities during urination, each animal was injected intraperitoneally (i.p.) with diuretic furosemide (40 mg/kg) to increase the frequency of voiding and the implanted fiber was directly connected to the fiber photometry system. Then, mice were moved into the experimental chamber that had a glass bottom and habituated for 20 min. A video recorder (Sony, Japan) was placed under the chamber to record videos (30 Hz with a spatial resolution of 1,280 × 720 pixels) of the urination events. The activities of the PMC-projecting VLPAG neurons and mouse behavior videos were detected simultaneously. Each recording session lasted approximately 40 min to 1 h. Fiber photometry recording data were sampled *via* acquisition software (LabVIEW platform, National Instruments, USA). According to the video data, we defined the onset of urine appearance from the mouse urinary meatus as the start of urination events, and defined no urine appearance at the urinary meatus as the termination of urination events (the urination offset).

### Cystometric Measurement in Freely Moving Mice

Animals were anesthetized with isoflurane (4% induction, 1%–1.5% maintenance throughout the surgery) and positioned on top of a heating pad. After local application of 100 μl lidocaine hydrochloride (2%) under the skin and lower abdominal muscle, we accessed the mouse bladder dome and exteriorized it *via* a 1-cm midline incision in the lower abdomen. A PE10 catheter (7 cm) was inserted and implanted into the bladder. Then, the other side of the PE10 catheter was removed through a 1 cm cut at the back of the mouse’s neck after local application of lidocaine hydrochloride (2%, 100 μl) under the skin. The surgical wound was sutured after verifying the seal and normal flow of the bladder catheter. Animals were rested for 3–5 days before cystometric measurement incorporating fiber photometry recording experiments were initiated. We used a high sensitivity pressure transducer (YPJ01H; Chengdu Instrument Factory, China) connected with the multi-channel physiological information recording device (RM6240; Chengdu Instrument Factory, China) to measure the bladder pressure. The injection pump (RWD404; RWD Technology Corp., Ltd., Shenzhen, China), pressure sensor, and adapter were connected to each other through a three-way tab. A PE50 tube was connected to the end of the adapter. Before cystometry recordings, the PE10 bladder catheter was moved into the other end of the PE50 tube. Freely moving animals were simultaneously measured for VLPAG neuronal activity and cystometry with bladders receiving an infusion of normal saline *via* the PE10 catheter at a constant speed of 30–50 μl/min. The cystometry data were collected by the commercially available acquisition software (Chengdu Instrument Factory, China).

### Perfusion and Immunohistochemistry

Immunohistochemistry staining was carried out as we previously described (Yao et al., 2018, 2019). After each experiment, mice were anesthetized with 10 ml/kg sodium pentobarbital (1%) and initially transcardially perfused using phosphate-buffered saline (PBS) or saline, followed by transcardial perfusion using 4% paraformaldehyde (PFA). Murine brains underwent dissected and were then fixed by placing samples in 4% PFA at 4°C for at least 24 h until sectioning. These brain samples were sliced into coronal slices or sagittal slices (40 μm) by use of a vibratome (NX50, Thermo). For tyrosine-hydroxylase (TH) immunohistochemistry staining, the primary antibody was rabbit anti-TH (1:200, Millipore, AB152) and a donkey anti-rabbit antibody conjugated with Alexa Fluor 647 was used as the secondary antibody (1:200, Invitrogen, A31573). Lastly, all sections were stained with DAPI (Beyotime, Shanghai, China, C1006). These brain slices were mounted on glass slides, cover-slipped, and imaged using a fluorescence microscope (BX53, Olympus) or a scanning confocal microscope (TCS SP5, Leica). Viral injection sites and fiber tip sites for each mouse was confirmed *via* *post hoc* images. Animals with improper locations of viral injection or fiber implantation were excluded from final data analyses.

### Data Analysis

Fiber photometry recording data were recorded at a 2,000 Hz sample rate and bladder pressure data were obtained at an 800 Hz sample rate. The optic-fiber recording data underwent low-pass filtering by use of a Savitzky-Golay filter with 3 as the polynomial order and 50 side points. We calculated the relative fluorescence change values, Δf/f = (f − f_baseline_)/f_baseline_), as Ca^2+^ transients, and f_baseline_ was defined as the minimum fluorescent signal detected when recording urination events. We considered the Ca^2+^ transient amplitude, which was three times higher than the standard deviation of the noise band, as a positive signal. To shuffle the GCaMP6f fluorescence data, we divided the GCamp6f fluorescence data into 10 segments that were then randomly assigned these segments with detected urination events. We counted the cell number of PRV531-labeled neurons and RV-labeled neurons manually.

### Statistical Analysis

All data in this article were represented as mean ± s.e.m‥ The dot plots in each figure are overlaid with the corresponding bar graphs. We used MATLAB programs (MathWorks, USA) to conduct our cross-correlation analyses and non-parametric statistical analyses. We used the Wilcoxon signed-rank test for the nonparametric paired data and the Wilcoxon rank-sum test for the two independent data. Statistical significance was determined as follows: ^***^P < 0.001, ^**^P < 0.01, ^*^P < 0.05; ns indicates no significant difference was detected.

## Results

### Anatomical Distribution of VLPAG Neurons Innervating the Bladder Detrusor in Brains From Adult Mice

**Figure 1:**
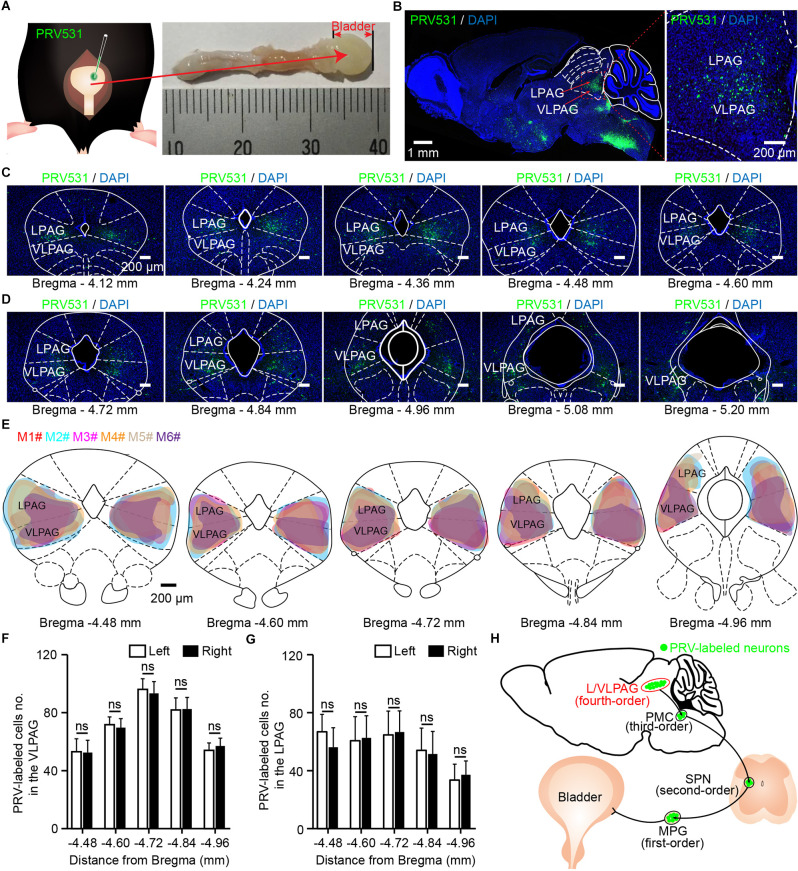
Identification of ventrolateral periaqueductal gray (VLPAG) neurons after PRV531 injection into the bladder wall. **(A)** Schematic for PRV531 injection into the bladder detrusor of adult mouse. **(B)** Sagittal brain section showing PRV531-infected VLPAG neurons from one mouse (red dotted box, enlarged on the right). **(C,D)** Serial coronal brain sections showing PRV531-infected neurons in the VLPAG of one mouse. **(E)**. Drawings of brain anatomy overlay showing major PRV531-labeled areas in the VLPAG/LPAG of adult mice (*n* = 6 mice, each mouse was indicated with different colors). **(F)** Quantification of cell numbers of VLPAG PRV531-labeled neurons on contralateral/ipsilateral sides (*n* = 6 mice). Wilcoxon signed-rank test, *P* > 0.05. **(G)** Quantification of cell numbers of LPAG PRV531-labeled neurons on contralateral/ipsilateral sides (*n* = 6 mice). Wilcoxon signed-rank test, *P* > 0.05. **(H)** Anatomical scheme of neural pathways innervating the bladder detrusor. SPN, sacral parasympathetic nuclei; MPG, major pelvic ganglion.

To confirm the distribution of VLPAG neurons that may participate in control of bladder and urination, we directly injected pseudorabies virus 531 (PRV531) expressing robust green fluorescent protein, a valuable retrograde trans-multisynaptic tracer (Jia et al., 2019) into the bladder detrusor of adult mice to label supraspinal neural circuits (Figure 1A). These mice were perfused when showing serious symptoms of infection and abnormal behaviors. Their average survival time after PRV531 injection was 103.9 h ± 3.0 h (mean ± s.e.m., *n* = 7 mice). Depending on the average survival time, PRV531 transported and infected transneuronally from one-order neurons to fourth-order neurons. In addition to the PAG, the major PRV531-labeled brain structures were found in the gigantocellular reticular nucleus (Gi), raphe nuclei, A5 noradrenaline cells, PMC, locus coeruleus (LC), red nucleus (Red N), lateral hypothalamic area (LH), paraventricular nucleus (PVN), medial preoptic area (MPOA), and cerebral cortex (data not shown). The above PRV531-labeled brain areas were similar to those reported in our previous study using PRV152 (Yao et al., 2018).

In the present experiment, we aimed to demonstrate the precise anatomical location of PRV531-labeled VLPAG neurons. According to the mouse brain atlas, morphological data of sagittal brain section (Figure 1B, *n* = 1 mouse) and serial coronal brain sections (Figures 1C,D) showed that PRV531-infected VLPAG neurons were bilaterally present in the whole VLPAG region (Bregma: −4.12–5.20 mm). A closer overlay analysis revealed that the majority of PRV531-labeled VLPAG neurons were located between Bregma −4.48 mm and Bregma −4.96 mm (Figure 1E, *n* = 6 mice). Quantification of retrogradely labeled cell numbers of the VLPAG in serial coronal brain sections showed no significant difference between the ipsilateral and contralateral sides (Bregma −4.48 to −4.96 mm, left side 53 ± 9–96 ± 7, right side 53 ± 8–93 ± 8, *P* > 0.05, Figure 1F, *n* = 6 mice). In addition, we also found the PRV531-infected LPAG neurons in these brain sections (Figures 1B–E). Quantification of PRV531-labeled neuron numbers of the LPAG in serial coronal brain sections showed no significant difference between the ipsilateral and contralateral sides (Bregma −4.48 to −4.96 mm, left side 34 ± 11–67 ± 12, right side 37 ± 10–67 ± 15, *P* > 0.05, Figure 1G, *n* = 6 mice). PRV-infected neurons in the VLPAG/LPAG should be fourth-order neurons (Figure 1H), according to our previous study (Yao et al., 2018). Altogether, these results indicate that both VLPAG and LPAG neurons innervate the bladder detrusor.

### Identification of VLPAG Neurons That Project to the PMC

**Figure 2:**
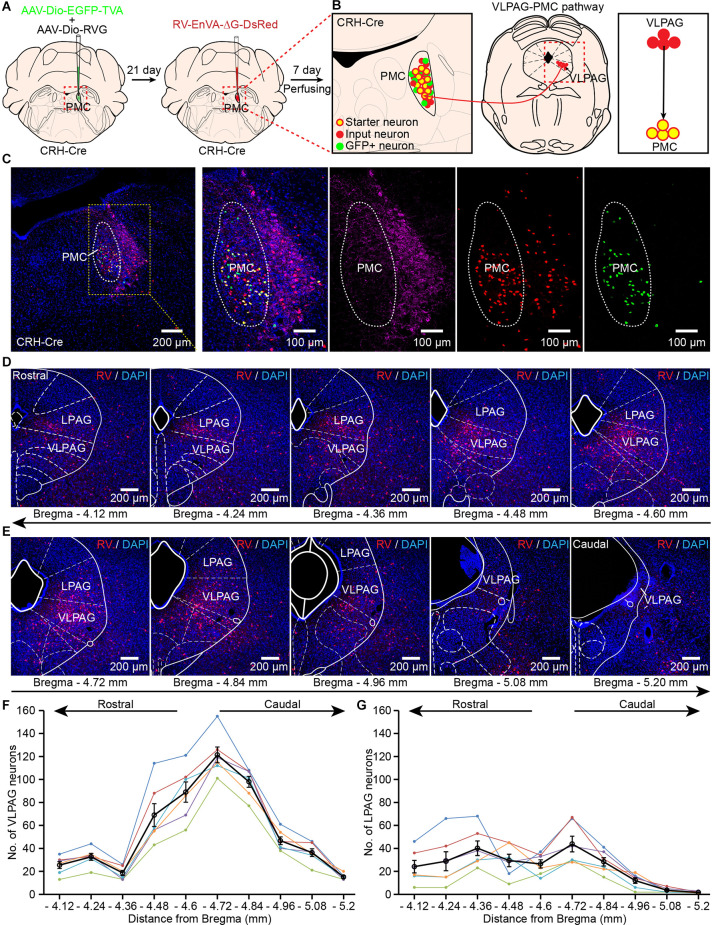
Identification of VLPAG neurons that project to PMC^CRH^ neurons. **(A)** Schematic of the experimental timeline and injection procedure for helper AAVs and RV into the pontine micturition center (PMC) of corticotropin releasing hormone (CRH)-Cre mice. **(B)** Strategy for retrograde trans-synaptic tracing of monosynaptic inputs from the VLPAG in PMC^CRH^ neurons using cell-type-specific RV system. **(C)** A representative image containing the PMC immunostained with tyrosine-hydroxylase (TH) (left). The starter neurons expressing helper AAVs and RV (yellow dotted box, enlarged on the right) were restricted to the unilateral PMC of one CRH-Cre mouse. **(D,E)** Serial coronal brain sections showing RV-labeled neurons in the VLPAG of one mouse. **(F)** Distributions of RV-labeled neurons in the whole ipsilateral VLPAG (Bregma: −4.12 to −5.20 mm, *n* = 6 mice, each mouse was indicated with different colors). The black line represents the average number of RV-labeled VLPAG neurons in each section. Data are presented as mean ± s.e.m. **(G)** Quantification of cell numbers of RV-labeled neurons in the ipsilateral LPAG (Bregma: −4.12 to −5.20 mm, *n* = 6 mice, each mouse was indicated with different colors). The black line represents the average number of RV-labeled LPAG neurons in each section. Data are presented as mean ± s.e.m.

PMC^CRH^ neurons were previously reported to be an important controller in bladder contractions (Hou et al., 2016; Ito et al., 2020). PMC^CRH^ neurons received direct input projections from PAG (Hou et al., 2016; Verstegen et al., 2019), but the cell body distribution of these PAG neurons has not been well-characterized. To further investigate the anatomical distribution of VLPAG neurons innervating the bladder detrusor that project to PMC^CRH^ neurons, we used cell-type-specific rabies virus (RV) system in CRH-Cre mice (Figure 2A). Firstly, we injected two Cre-dependent helper adeno-associated viruses (AAV-Dio-RVG and AAV-Dio-EGFP-TVA) into the right PMC (Figure 2A). Then, we injected RV (RV-EnvA-ΔG-DsRed) into the same area 3 weeks after the first injection (Figure 2A). One week later, we perfused these mice and obtained their brains (Figure 2A).

The starter neurons (yellow cells) were restricted to the right PMC and co-labeled by helper AAV and RV (Figures 2B,C). Retrogradely labeled monosynaptic input neurons from the VLPAG only expressed RV-DsRed (Figures 2B,D,E). The representative serial coronal brain sections of the VLPAG (Figures 2D,E) revealed that RV-labeled PAG neurons were widely distributed in the whole ipsilateral VLPAG (Bregma: −4.12 to −5.20 mm). The quantification of RV-labeled neuronal number in the ipsilateral VLPAG showed that most of RV-labeled VLPAG neurons were also located in the medial and caudal regions of the VLPAG (Bregma −4.48 to −4.96 mm, Figure 2F, *n* = 6 mice). The average number of cells in the VLPAG ranged from 15 ± 1 (Bregma −5.20 mm) to 121 ± 7 (Bregma −4.72 mm). We also found the RV-labeled LPAG neurons in these serial coronal brain sections (Figures 2D,E). Quantification of RV-labeled cell numbers in the ipsilateral LPAG (Figure 2G) showed that the average number of neurons ranged from 2 ± 0 (Bregma −5.20 mm) to 44 ± 7 (Bregma −4.72 mm). These findings confirm the accurate distribution of VLPAG neurons that project to PMC^CRH^ neurons. Moreover, our results suggest that LPAG neurons project to PMC^CRH^ neurons directly. We used the above location of the VLPAG for the following optical-fiber recording experiments.

### Population Ca^2+^ Signals of VLPAG Neurons Projecting to the PMC Highly Correlate With Urination

**Figure 3:**
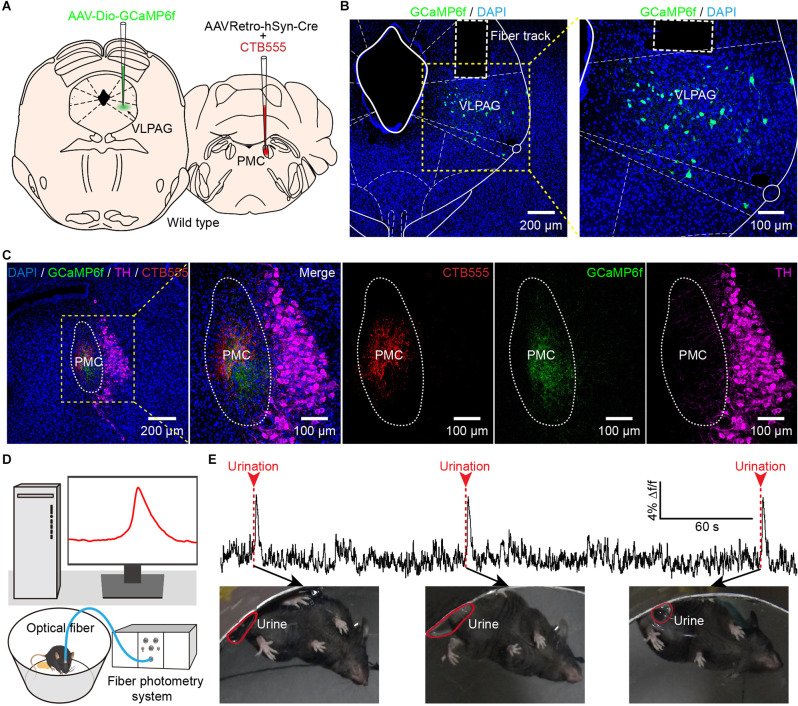
*In vivo* fiber photometry recording of the activity of PMC-projecting VLPAG neurons during urination in freely moving mice. **(A)** Schematic of AAVRetro-hSyn-Cre injection into the PMC and AAV-Dio-GCaMP6f injection into the VLPAG. **(B)** The representative picture shows that PMC-projecting VLPAG neurons are labeled with GCaMP6f (yellow dotted box, enlarged image on the right). **(C)** Confocal images from the same mouse in **(B)** of CTB555 (red) and the axon terminals of GCaMP6f-labeled VLPAG neurons (green) in the PMC. The locus coeruleus (LC) is stained with TH (magenta). **(D)** Schematic of fiber photometry recording experiment during urination in freely moving mouse. **(E)** An example trace for GCaMP6f fluorescence change of PMC-projecting VLPAG neurons during urination events. Red arrows and dotted bars indicate initiation of three urination events.

The VLPAG is considered to be the major PAG column for bladder control *via* its direct projection to the PMC (Zare et al., 2019). The role of LPAG neurons in controlling bladder function is rarely reported. Therefore, we only explored the real-time Ca^2+^ activity of PMC-projecting VLPAG neurons in response to natural urination events through monitoring activities of these VLPAG neurons and urination events in freely behaving mice simultaneously in the following experiment. We expressed genetically encoded Ca^2+^ indicator GCaMP6f in PMC-projecting VLPAG neurons by injecting AAVRetro-hSyn-Cre mixed with CTB555 into the right PMC and AAV encoding Cre-dependent GCaMP6f (AAV-Dio-GCaMP6f) into the right VLPAG (Figure 3A). After three weeks, we implanted a 200 μm diameter optical fiber above the VLPAG and applied an optical fiber-based Ca^2+^ signal recording system to specifically monitor the Ca^2+^ activity of GCaMP6f-labeled PMC-projecting VLPAG neurons during urination (Figures 3B,D). Representative images revealed that the injection site of AAVRetro-hSyn-Cre mixed with CTB555 and axon terminals of these GCaMP6f-labeled VLPAG neurons were only restricted to the PMC (Figure 3C). We observed large increases in calcium activity of PMC-projecting VLPAG neurons at each time of urination (Figure 3E).

**Figure 4:**
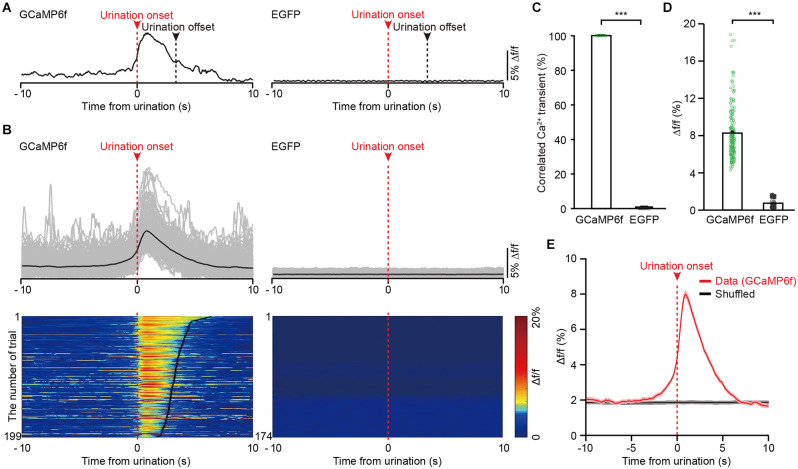
The activities of VLPAG neurons projecting to the PMC correlate with urination in freely moving mice. **(A)** Example individual recording traces from GCaMP6f-labeled (left) and EGFP-labeled (right) PMC-projecting VLPAG neurons. Dashed red lines and red arrows indicate urination onsets. Dashed black lines indicate urination offsets. **(B)** Top, overlay of all trials in the GCaMP6f-labeled group (199 urination events, *n* = 8 mice) and the EGFP-labeled control group (174 urination events, *n* = 7 mice). Bottom, heatmaps of individual recording traces aligned to urination event onsets. The bold black lines indicate urination offsets in the left heatmap. **(C)** Quantification of the percentage of Ca^2+^ transients correlated with urination in the GCaMP6f-labeled group and EGFP-labeled control group. **(D)** Quantification of amplitudes of all trials in GCaMP6f-labeled group and EGFP-labeled control group. Wilcoxon rank-sum test, ^***^P < 0.001. **(E)** Averaged Ca^2+^ signal in GCaMP6f-labeled PMC-projecting VLPAG neurons aligned to the urination onset or to the shuffled urination onset.

We observed that Ca^2+^ signals of GCaMP6f-labeled PMC-projecting VLPAG neurons were sustained throughout the entire duration of urination events (Figures 4A,B). We also found that the onsets of Ca^2+^ signals of GCaMP6f-labeled PMC-projecting VLPAG neurons were earlier than the urination event onsets in 181/199 trials (91% of trials), suggesting that the activities of these VLPAG neurons may contribute to the initiation of the urination events (Figures 4A,B, Supplementary Figures 1A,B). A robust increase in GCaMP6f fluorescence of PMC-projecting VLPAG neurons started 500 ms (505 ± 32 ms) before the urination event onsets (Supplementary Figure 1A). However, no change in fluorescence of EGFP-labeled PMC-projecting VLPAG neurons during urination was detected (Figures 4A–D, GCaMP6f-labeled group, max Δf/f = 8.25% ± 0.20%; EGFP-labeled control group, max Δf/f = 0.74% ± 0.04%; *P* < 0.001), suggesting that the urination-related GCamp6f signals were not a result of movement artifacts. Across all the recording trials, each urination event corresponded to the Ca^2+^ signal of GCaMP6f-labeled PMC-projecting VLPAG neurons (Figures 4B,C). Further analysis revealed that the urination-correlated Ca^2+^ activity was absent in randomly chosen intervals (shuffled data, Figure 4E). Thus, our data indicates that the population Ca^2+^ activity of PMC-projecting VLPAG neurons highly correlates with urination.

### Activity of VLPAG Neurons Projecting to the PMC Increases During Bladder Contraction

**Figure 5:**
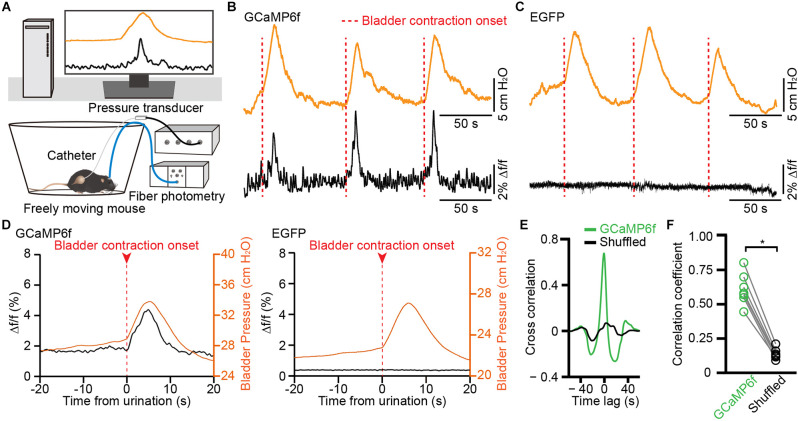
Population Ca^2+^ transients of VLPAG neurons projecting to the PMC correlate with increased bladder pressure in freely moving mice. **(A)** Schematic of fiber photometry recording experiment during cytometry in freely moving mouse. **(B,C)** Representative time-locked GCaMP6f or EGFP fluorescence (black) and bladder pressure (orange) traces from GCaMP6f-labeled **(B)** and EGFP-labeled **(C)** PMC-projecting VLPAG neurons. Dashed red lines indicate bladder contraction onsets. **(D)** Averaged bladder pressure (orange) and time-locked fluorescence (black) trace from GCaMP6f-labeled (left) group (*n* = 63 events from seven mice) and EGFP-labeled (right) control group (*n* = 59 events from seven mice). Averaged fluorescence trace aligned to the onset of bladder contractions. **(E)** A representative example of cross-correlation between Ca^2+^ signals of VLPAG neurons projecting to the PMC (green) and bladder pressure from one mouse compared to the shuffled data (black). **(F)** Quantification of cross-correlation coefficients. *n* = 7 mice. Wilcoxon signed-rank test, ^*^P < 0.05.

To further investigate whether bladder contraction coincides with the calcium signals of PMC-projecting VLPAG neurons, we simultaneously measured bladder pressure by use of cystometry and GCaMP6f fluorescence changes in freely moving animals (Figure 5A). We determined that each instance of increased activity of GCaMP6f-labeled PMC-projecting VLPAG neurons was associated with a spike in bladder pressure (Figures 5B–D, *n* = 7 mice). We did not identify an increase in fluorescence in EGFP-labeled PMC-projecting VLPAG neurons during bladder contraction (Figures 5C,D, *n* = 7 mice). Averaging Ca^2+^ signals synced to the onset of bladder contractions showed a sharp increase in GCaMP6f fluorescence when the initial surge of bladder pressure (Figure 5D). Time-locked Ca^2+^ signals of GCaMP6f-labeled PMC-projecting VLPAG neurons started 68 ms on average before the bladder contraction onsets (Figure 5D). The onsets of Ca^2+^ signals of these PMC-projecting VLPAG neurons were earlier than the bladder contraction onsets in 34/63 trials (54% of trials). Each bladder contraction was 100% associated with Ca^2+^ activity of VLPAG neuronal populations projecting to the PMC. Cross-correlation analysis between GCaMP6f signals and the measured bladder pressure indicated that the mean peak’s correlation coefficient for GCaMP6f data was found to be significantly higher compared to the shuffled data (Figures 5E,F, GCaMP6f data, 0.61 ± 0.04; shuffled data, 0.14 ± 0.01; *P* < 0.05). Our findings suggest that these Ca^2+^ signals from PMC-projecting VLPAG neurons have a significant association with bladder contraction.

### VLPAG Neurons That Project to the PMC Receive Inputs From Many Upstream Brain Areas

**Figure 6:**
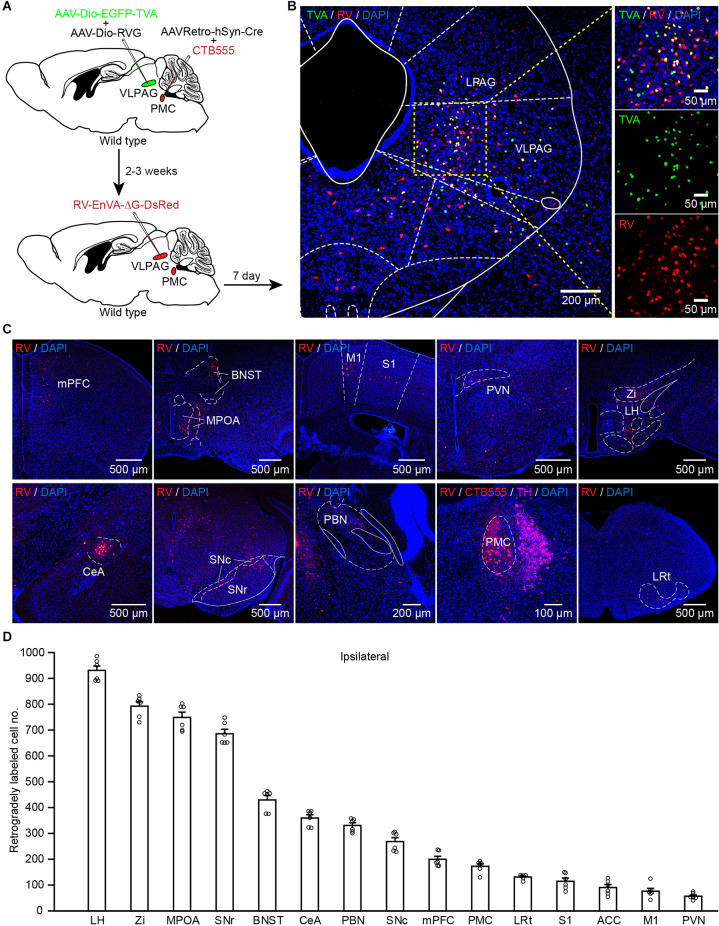
PMC-projecting VLPAG neurons receive monosynaptic inputs from upstream brain areas. **(A)** Schematic of the experimental timeline and injection procedure. **(B)** Representative images showing starter neurons expressing helper AAVs and RV (yellow, enlarged in the right) restricted to the unilateral VLPAG. **(C)** Representative coronal sections of RV-labeled neurons distributed in many ipsilateral upstream brain regions of PMC-projecting VLPAG neurons. **(D)** Quantification of the total number of RV-labeled neurons in each ipsilateral upstream brain region of PMC-projecting VLPAG neurons.

The above data indicate that PMC-projecting VLPAG neurons are activated during urination and bladder contractions. We wondered which brain regions could direct input to PMC-projecting VLPAG neurons. To unravel monosynaptic inputs to VLPAG neurons that ultimately project to the PMC, a RV-based retrograde trans-synaptic tracing was used. We injected AAVRetro-hSyn-Cre mixed with CTB555 into the right PMC and two Cre-dependent helper AAV (presented above) into the right VLPAG (Figure 6A). At 2–3 weeks after the first injection, we injected RV-EnvA-ΔG-DsRed into the right VLPAG (Figure 6A). Representative expanded images were obtained 1 week later and showed that starter neurons (yellow cells) were restricted to the VLPAG (Figure 6B).

Coronal brain images from a representative mouse revealed that major input neurons to PMC-projecting VLPAG neurons were observed in many upstream brain areas (on the ipsilateral side), including prefrontal areas, primary motor cortex (M1), primary somatosensory cortex (S1), bed nucleus of the stria terminalis (BNST), MPOA, PVN, LH, zona incerta (Zi), central amygdalar nucleus (CeA), substantia nigra pars compacta (SNc), substantia nigra pars reticulata (SNr), parabrachial nucleus (PBN), PMC, and lateral reticular nucleus (LRt; Figure 6C). Then we performed a statistical analysis on the input cell numbers obtained from the above major brain regions on the ipsilateral side of the injection site (Figure 6D, *n* = 6 mice). The average number of RV-labeled neurons throughout the above brain regions (Figure 6D) ranged from 57 ± 5 (PVN) to 931 ± 16 (LH). These results show a clear distribution of input across the whole brain to PMC-projecting VLPAG neurons.

## Discussion

The physiological purpose of urination is to maintain fluid balance and eliminate urine outside the body. The current working model of urine elimination is that PAG neurons consequently activate the PMC *via* direct projections from the PAG to initiate urination reflex after receiving the decision to void made by upstream brain structures (Fowler and Griffiths, 2010; Kitta et al., 2015). These PMC-projecting PAG neurons likely act as a relay point for transmitting pro-urination signals to the PMC. It has already been confirmed that PAG neurons (especially VLPAG neurons) and PMC neurons have anatomical and functional synaptic connections (Verstegen et al., 2019). Nevertheless, the exact locations of PMC-projecting VLPAG neurons have not been demonstrated. Many previous articles have shown that PMC neurons are only active during urination and bladder contraction (Hou et al., 2016; Verstegen et al., 2019; Yao et al., 2019). However, the activities of PMC-projecting VLPAG neurons during urination or the urination cycle and whole-brain inputs of these VLPAG neurons remains poorly understood.

In recent decades, powerful tools have been developed in the field of neuroscience, such as those utilizing viruses (Tervo et al., 2016) and fiber photometry (Adelsberger et al., 2005; Gunaydin et al., 2014), which have made it possible to address these issues. Here, using PRV-mediated retrograde tracing technique, we found that both VLPAG and LPAG neurons innervate the bladder detrusor. Then, we identified the location of VLPAG neurons that project to PMC^CRH^ neurons innervating the bladder detrusor. In addition, we noticed that many LPAG neurons projected to PMC^CRH^ neurons, suggesting that the LPAG may also play a role in bladder control. In the current work, we mainly focused on the activities of PMC-projecting VLPAG neurons during urination. Subsequently, we specifically labeled VLPAG neurons projecting to the PMC with GCaMP6f and confirmed that population activities of these VLPAG neurons highly correlated with voiding and bladder contraction. We also observed that these bladder contraction-related VLPAG neurons received extensive inputs from multiple higher brain areas. Our results suggest that PMC-projecting VLPAG neurons are important elements in bladder control and provide additional support for the above working hypothesis. Notably, we did not monitor activities of PMC-projecting LPAG neurons during urination, although LPAG neurons were also labeled by PRV and RV. The exact function of PMC-projecting LPAG neurons in the control of bladder remains to be investigated in future.

Previous work report that urination-related activities of PAG neurons are detected by *in vivo* single-unit recordings during cystometry in supracollicular decerebrated animals (Liu et al., 2004). Their results show several types of PAG neurons based on their different firing patterns, including phasic micturition neurons, tonic micturition neurons, phasic storage neurons, and tonic storage neurons (Liu et al., 2004; de Groat et al., 2015). Using functional brain imaging and cystometry, some studies have also indicated an increase in activity of the PAG during urine storage and further enhanced activity of the PAG during voiding (Tai et al., 2009; Wong et al., 2015). Some other studies explore the activation of PAG neurons evaluated by c-Fos after chemical irritation or electro-stimulation of the bladder (Mitsui et al., 2003; Meriaux et al., 2018). Different patterns of neural activity in the PAG may correspond to complex functions of PAG neurons in urine storage and urination. Here, using fiber photometry accompanied by cystometry, we were able to record the circuit-specific VLPAG neurons afforded by AAVs (AAV-Dio-GCaMP6f and AAVRetro-hSyn-Cre) during spontaneous urination or bladder contraction in freely moving animals at a higher temporal resolution. Our results only showed PMC-projecting VLPAG neurons were active during urination and bladder contraction, similar to phasic micturition neurons in the PAG. Any activities of PMC-projecting VLPAG neurons during urine storage were not observed. It is suggested that these VLPAG neurons are likely different from those PAG neurons receiving bladder afferent information. So, neuronal subpopulations in the PAG involved in bladder storage function still need to be further investigated.

Urination patterns in mice are affected by internal states, experience, and external environment (Hou et al., 2016), including hormone homeostasis, nociception, social dominance, stress, etc. (Mukhopadhyay and Stowers, 2020). In addition, in this study, we applied continuous saline infusion into the bladder to induce urination cycles, which is not exactly the same as the physiological urination. This manipulation may often induce a reflexive urination (Nishijima et al., 2012; Yamamoto et al., 2020) and thus may not require the drive from VLPAG neurons (de Groat et al., 2015). These possibilities probably explain why we observed that the onsets of activities of PMC-projecting VLPAG neurons prior to the bladder contraction only occurred in half of trials. Therefore, these results indicate that PMC-projecting VLPAG neurons contribute to the initiation of the bladder contraction in some situations (e.g., urine scent marking, voiding at an appropriate location) (Zare et al., 2019; Mukhopadhyay and Stowers, 2020) but contribute to sustain bladder detrusor contractions in other situations (e.g., intense fear state, stressful state; Stone et al., 2011).

Neuroanatomical tracing studies demonstrate that some higher regions send afferents to the PAG, including the medial prefrontal cortex, hypothalamus, and amygdala (Beart et al., 1990). Connectivity analysis in functional brain imaging studies uncovers that the PAG is connected with many brain structures during voiding (Fowler and Griffiths, 2010; Zare et al., 2019). Our results using an RV-based tracing strategy showed that urination-related PMC-projecting VLPAG neurons received dense inputs from multiple urination-related higher brain areas, such as the MPOA, medial prefrontal cortex, and LH. Previous articles have provided evidence supporting that these regions govern urine marking behavior. Chemogenetic inhibition of MPOA GABAergic neurons impairs urine marking and urination (Hou et al., 2016). The MPOA likely acts as a coordinating node to modulate voiding for territorial demarcation. Dominant male mice release their urine throughout the cage (Desjardins et al., 1973; Hou et al., 2016). The medial prefrontal cortex is a critical region for modulating the social hierarchy of rodents (Wang et al., 2011). Chemogenetic activation or inhibition of LH neurons also influences urination patterns (Hyun et al., 2021). Thus, PMC-projecting VLPAG neurons likely regulate urination under the control of urination-related higher brain areas.

Bladder dysfunction is a common clinical characteristic of various nervous system diseases, such as Parkinson’s disease (PD), multiple sclerosis (MS), and Wernicke’s encephalopathy (Benarroch, 2010; Zare et al., 2019). Brain function imaging research shows that the PAG is significantly more active during detrusor overactivity in PD patients (Kitta et al., 2006). Ablating PAG dopaminergic neurons impairs urination patterns in an animal model of PD (Zare et al., 2019). Bladder disability scores highly correlate with damage size of urination-related brain regions including the PAG in MS patients (Charil et al., 2003) and PAG lesions occurs in 18% of these patients (Papadopoulou et al., 2014). Wernicke’s encephalopathy is caused by vitamin B1 deficiency leading to neuronal degeneration in the PAG (Sakakibara et al., 1997). In this work, we have shown the exact distribution of urination-related VLPAG neurons, which provides a target region for treatment of these nervous system diseases.

In summary, our findings firstly demonstrate that PMC-projecting VLPAG neurons receiving extensive whole-brain inputs are involved in bladder control and their activities highly correlate with bladder contraction. Our work presents new insight into the neuronal circuits involved in controlling bladder function and presents a potential therapeutic brain node for neurogenic bladder dysfunction.

## Data Availability Statement

The raw data supporting the conclusions of this article will be made available by the authors, without undue reservation.

## Ethics Statement

The animal study was reviewed and approved by the Third Military Medical University Animal Care and Use Committee.

## Author Contributions

JYao, XC, and JYan contributed to the design of the study and interpretation of the data. YR, ZG, XianL, XingL, JL, DL, and JZ performed the experiments and acquired the data. JYao, YR, SL, and ZG processed and analyzed the data. JYao, XC, and JYan wrote the manuscript with help from all the other authors. All authors contributed to the article and approved the submitted version.

## Conflict of Interest

The authors declare that the research was conducted in the absence of any commercial or financial relationships that could be construed as a potential conflict of interest.

## Publisher’s Note

All claims expressed in this article are solely those of the authors and do not necessarily represent those of their affiliated organizations, or those of the publisher, the editors and the reviewers. Any product that may be evaluated in this article, or claim that may be made by its manufacturer, is not guaranteed or endorsed by the publisher.
