# Supplementary material for: Ventrolateral Periaqueductal Gray Neurons Are Active During Urination
Source: Front Cell Neurosci. 2022 Jun 23;16:865186. doi: 10.3389/fncel.2022.865186 (PMC9259957; doi:10.3389/fncel.2022.865186)
Supplement: Supplementary file 1 [file Data_Sheet_1.DOCX]

Supplementary Material

## Supplementary Figures


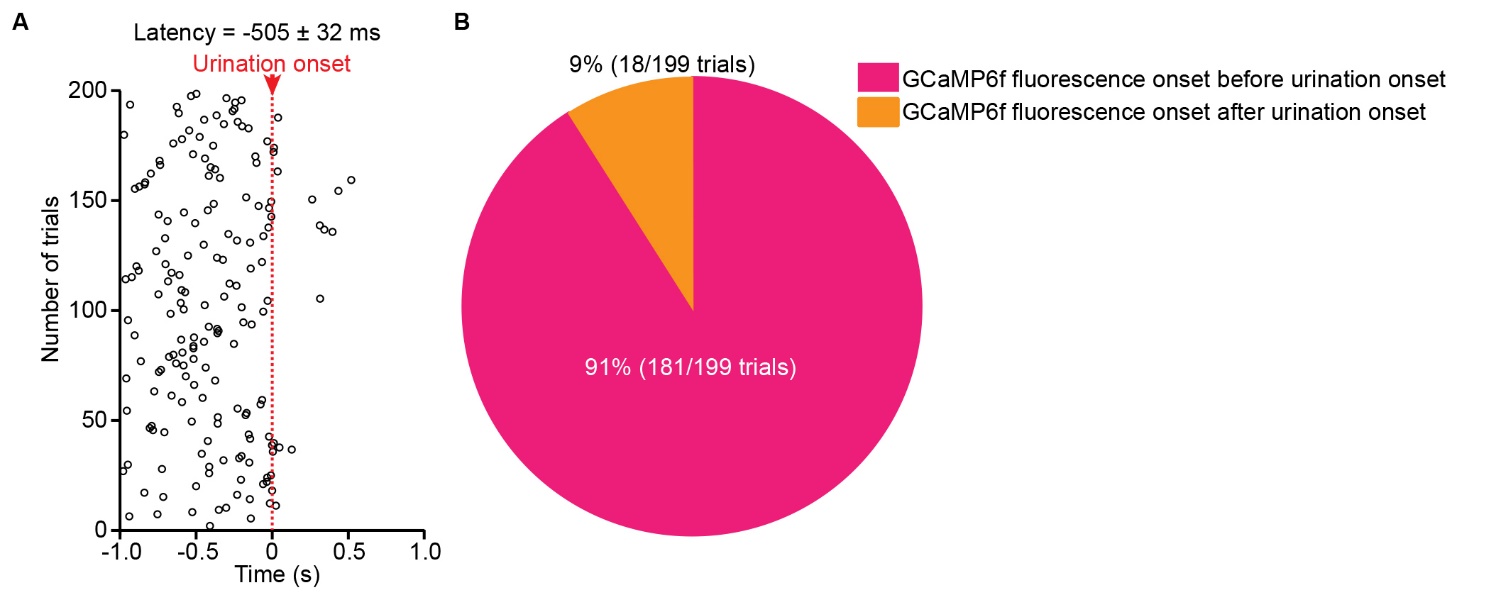


**Supplementary Figure 1.** The time interval between the Ca^2+^ signal starting point and the urination onset. **(A)** Onsets of Ca^2+^ signals of PMC-projecting VLPAG neurons plotted relative to the onset of urination events. **(B)** The onsets of Ca^2+^ signals were earlier than the urination event onsets in 181/199 trials (91%).
